# Predictive factors for early hypothyroidism following the radioactive iodine therapy in Graves’ disease patients

**DOI:** 10.1186/s12902-020-00557-w

**Published:** 2020-05-29

**Authors:** Rui-Ting Hu, De-Shan Liu, Bin Li

**Affiliations:** 1 Department of Traditional Chinese Medicine, Qilu Hospital, Cheeloo College of Medicine, Shandong University, Jinan, 250012 Shandong China; 2 Department of Endocrinology, Hospital of Traditional Chinese Medicine of Linyi City, Linyi, 276002 Shandong China; 3 Department of Nuclear Medicine, Linyi People’s Hospital, Cheeloo College of Medicine, Shandong University, Linyi, 276000 Shandong China

**Keywords:** Graves’ disease, Radioactive iodine, Therapy, Early hypothyroidism

## Abstract

**Background:**

Radioactive iodine (RAI) therapy is an important treatment option for Graves’ disease (GD), the main side effect of RAI treatment is hypothyroidism, and the factors resulting in hypothyroidism are still controversial. The purpose of this retrospective study was to clarify the possible risk factors of early hypothyroidism after RAI therapy in Graves’ disease.

**Methods:**

We reviewed 312 GD patients treated with RAI between January 2017 to December 2018, collected the potential risk factors, and analyzed the relationship between these variables and early hypothyroidism.

**Results:**

After 6 months’ follow-up, 218 (69.87%) patients were evaluated as early hypothyroid. Male gender, shorter duration of disease, smaller thyroid weight, lower 2-h radioactive iodine uptake (RAIU), 6-h RAIU, 24-h RAIU and 6/24-h uptake ratio, lower administered dosages were significantly associated with early hypothyroidism. Logistics regression analysis showed that male gender, smaller thyroid weight and lower 6-h RAIU were associated with early hypothyroidism. Multi-factors combined ROC curve analysis suggested that the predictive power of male gender, smaller thyroid weight and lower 6-h RAIU for early hypothyroidism was 0.711.

**Conclusions:**

Our results show that RAI is an effective therapy for GD and most of the cured patients became to hypothyroid within 6 months. Male gender, smaller thyroid weight and lower 6-h RAIU are the main risk factors for early hypothyroidism.

## Background

Hyperthyroidism is a clinical syndrome caused by increased thyroid hormone in blood, it can lead to multiple complications, including cardiac, hepatic and hematologic system complications. More than 80% of the hyperthyroidism are caused by Graves’ disease (GD), and 3% of women and 0.5% of men may suffer GD in their lifetime [1]. The incidence is 20 to 30 cases per year per 100,000 persons [2]. The incidence peaks between 30 and 60 years of age, but people can be affected at any age [1, 2]. Common symptoms are palpitations, fatigue, tremor, anxiety, disturbed sleep, weight loss, heat intolerance, sweating, and polydipsia [3].

GD can be treated by anti-thyroid drugs (ATD), radioactive iodine (RAI) or thyroidectomy [4, 5]. ATD have lower risk of permanent hypothyroidism compared to RAI or thyroidectomy, but they are likely to cause frequently mild side effects and some rare but severe side effects, such as rash, vasculitis, agranulocytosis and acute hepatonecrosis, so the patients taking medications requires frequent hematological examinations [6]. Commonly, after 12–18 months’ drug treatment, the risk of recurrence is around 50% [7, 8]. Compared to ATD, RAI is a definitive treatment of hyperthyroidism and can improve hyperthyroidism quickly [9, 10]. Due to its low cost and high efficiency, lots of patients are tend to use RAI therapy to cure GD all over the world [11, 12].

Although the side effects of RAI are rare, mild and transient, it could result in hypothyroidism frequently. The aim of this study is to examine the occur of early hypothyroidism in GD patients treated with RAI in our center between January 2017 to December 2018, and to identify the significant risk factors influencing the occurrence of early hypothyroidism.

## Methods

### Patients

**Fig. 1:**
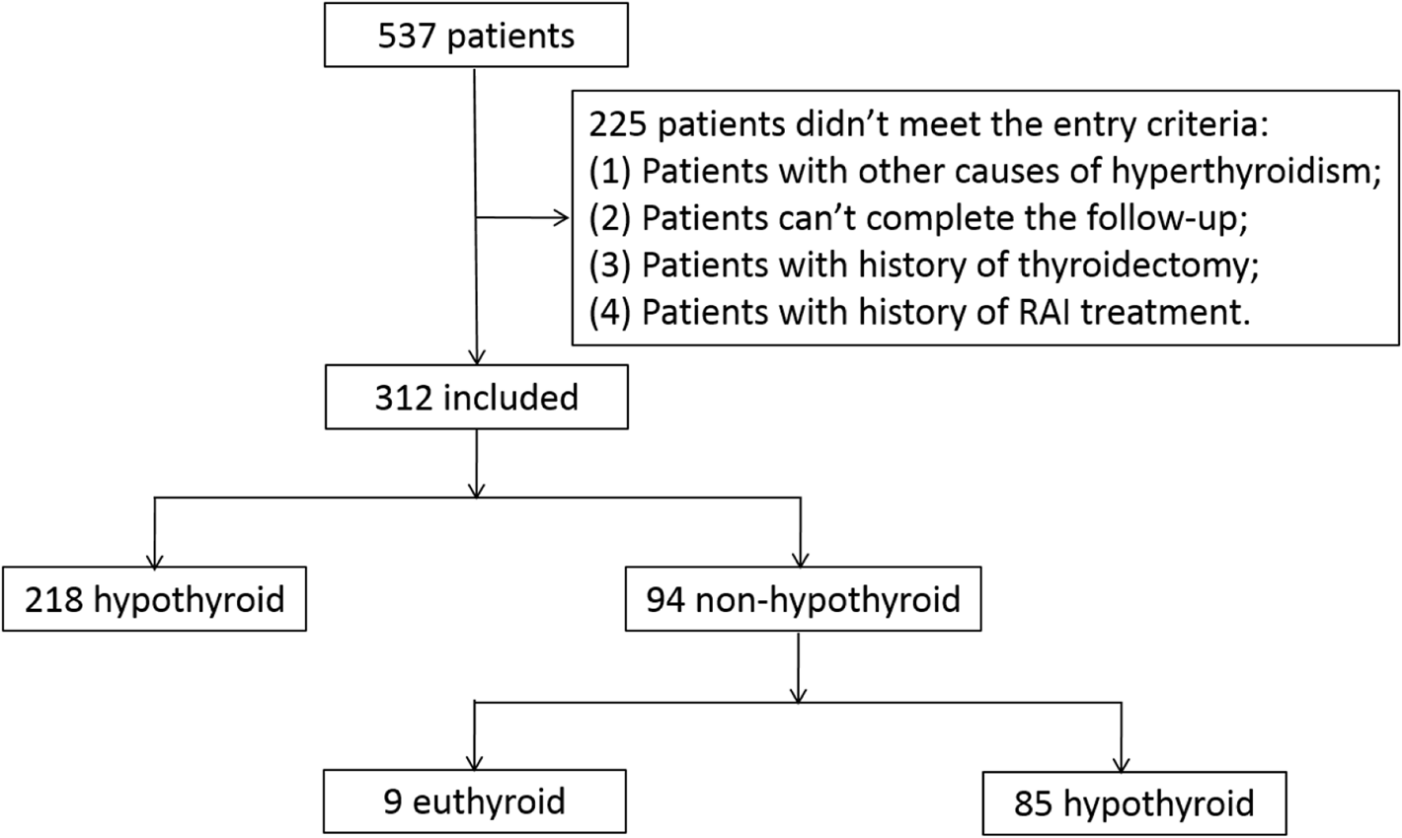
The Patient selection procedure and results after RAI treatment

As shown in Fig.1, in this retrospective study, 537 GD patients treated with RAI between January 2017 to December 2018 at Linyi People’s Hospital Affiliated to Shandong University (Linyi, China) were collected and reviewed. The inclusion criteria including: (1) Diffuse enlargement of the thyroid gland, symptoms of high metabolism including excitable, palpitation, hands tremble or emaciation; (2) suppressed serum thyrotropin (TSH) (< 0.55 μU/mL), elevated serum free triiodothyronine (FT3) (> 6.5 pmol/L), free thyroxine (FT4) (> 22.7 pmol/L) and TSH receptor antibody (TRAb). The exclusion criteria including: (1) Patients with other causes of hyperthyroidism, such as toxic multinodular goiter and single toxic adenoma; (2) Patients can’t complete the follow-up within 6 months; (3) Patients with history of thyroidectomy; (4) Patients with history of RAI treatment. After the evaluation, 225 patients didn’t meet the entry criteria, so the final number of the patients included in this study was 312. The following risk factors were collected and analyzed, including gender, age, duration of disease, weight of thyroid, ATD therapy history, FT3, FT4, TSH, TRAb, thyroperoxidase antibody (TPOAb), 2-h RAIU, 6-h RAIU, 24-h RAIU, 6/24-h uptake ratio and the administered dosages according to the calculated dosage method. The study was approved by the ethics committee of Linyi People’s Hospital Affiliated to Shandong University and was performed in accordance with the guidelines and regulations. Informed written consent was obtained from the all patients.

### Radioactive iodine uptake measurements

Before the test, all the patients need to stop taking iodine-rich foods, iodine-containing drugs and ATD for 2 weeks. During the examination, the patient took 131I solution 370 kBq (10 μCi) in the morning without eating any food, and kept fasting for an extra hour after taking it. Then the thyroid radioactivity counts at 2, 6, and 24 h after oral administration of the 131I solution were measured [13]. RAIU was calculated according to the following formula:

$$ \mathrm{RAIU}\kern0.5em \left(\%\right)=\frac{\mathrm{the}\;\mathrm{a}\mathrm{ctivity}\kern0.17em \mathrm{over}\kern0.17em \mathrm{the}\kern0.17em \mathrm{thyroidregion}-\mathrm{background}\;}{\mathrm{the}\;\mathrm{a}\mathrm{ctivity}\kern0.17em \mathrm{measured}\kern0.17em \mathrm{from}\;\mathrm{a}\;\mathrm{standard}\kern0.17em \mathrm{containing}-\mathrm{background}}\times 100\% $$

### Thyroid volume and weight estimation

The thyroid volume is measured by thyroid ultrasonography. In short, the patient lies on the back in the examination bed, fully exposing the neck, then the size of thyroid was measured independently by the two same experienced ultrasonic technicians using Ultrasound System (GE Vingmed Ultrasound AS, Horten, Norway). The thyroid weight was calculated with the following formula [14]:

$$ {\displaystyle \begin{array}{l}\mathrm{Thyroidweight}\;\left(\mathrm{g}\right)=0.479\times \Big[\mathrm{lenght}\;\left(\mathrm{cm}\right)\times \mathrm{width}\;\left(\mathrm{cm}\right)\times \mathrm{thickness}\;\left(\mathrm{cm}\right)\mathrm{of}\kern0.17em \mathrm{left}\;\\ {}\mathrm{lobe}+\mathrm{length}\;\left(\mathrm{cm}\right)\times \mathrm{width}\;\left(\mathrm{cm}\right)\times \mathrm{thickness}\;\left(\mathrm{cm}\right)\mathrm{of}\kern0.17em \mathrm{right}\kern0.17em \mathrm{lobe}\Big]\end{array}} $$

### Radioactive iodine dose calculation

The dose of RAI was calculated based on thyroid weight and RAIU as previously described [15]. In short, it was calculated according to the following formula:

$$ \mathrm{Oral}\;\mathrm{RAI}\;\mathrm{dose}\left(\mathrm{mCi}\right)=\frac{\mathrm{thyroidweight}\;\left(\mathrm{gram}\right)\times 0.1\left(\mathrm{mCi}/\mathrm{gram}\right)}{24\mathrm{h}\;\mathrm{RAI}\mathrm{U}\;\left(\%\right)} $$

### Radioactive iodine therapy

All the patient need to stop ATD and iodine-containing drugs for 2 weeks prior to RAI treatment, and followed a low-iodine diet for 2 weeks. After fasting and water deprivation for 8 h, all the patients took RAI orally in the morning. After taking RAI, the patients need to continue fasting and water deprivation for extra 2 h to avoid the effects of food on iodine absorption.

### Follow-up

Evaluation of the response was based on clinical manifestation and laboratory results, which was performed at each visiting per month after the treatments. The outcomes of RAI were classified as follows: (a) persistent hyperthyroidism—increased hormone levels (FT3 > 6.5 pmol/L, FT4 > 22.7 pmol/L, TSH < 0.55 μU/mL) and symptoms of hyperthyroidism after 6 months’ follow-up; (b) euthyroidism—normal hormone levels (FT3 3.5 to 6.5 pmol/L, FT4 11.5 to 22.7 pmol/L, TSH 0.55 to 4.78 μU/mL), and no symptoms of hyperthyroidism after 6 months’ follow-up; and (c) hypothyroidism—decreased hormone levels (FT3 < 3.5 pmol/L, FT4 < 11.5 pmol/L, TSH > 4.78 μU/mL) and symptoms of hypothyroidism within 6 months’ follow-up.

### Statistical analysis

The continuous variables were presented as mean ± standard deviation and non-continuous variables were presented as median (min, max). For normally distributed variables, the differences between the two groups were analyzed by the unpaired t-test. For non-normally distributed variables, the differences between the two groups were analyzed by the Mann–Whitney U test. Categorical variables were reported in term of frequency and percentage. Categorical outcomes were analyzed by chi-square or Fisher exact test, as appropriate. Logistic regression analysis was used to evaluate the impact of particular parameters contributed to the outcome of hypothyroidism after the RAI treatment. The threshold for statistical significance was set to *p* < 0.05. All statistical analyses were performed using SPSS software (Version 22.0; SPSS Inc., Chicago, IL, USA).

## Results

### The basic clinical characteristics of the patients in this study

As shown in Table 1, out of the 312 patients, 258 (82.7%) were female and the remaining 54 (17.3%) were male. The female to male ratio was 4.78:1, showing a higher incidence of Graves’ hyperthyroidism in female than male subjects. The age range of the patients in our study was 18–75 years, and the average age was 45.07 years. The median duration of the disease was 6 months, and the median weight of the thyroid was 52.21 g. Before switched to the RAI therapy, 42 male patients and 205 female patients were initially treated with ATD. Five male patients and 26 female patients took propylthiouracil and the rest patients took methimazole. The median levels of FT3, FT4 and TSH were 22.08 pmol/L, 51.68 pmol/L and 0.005 uIU/mL, respectively. The median levels of TPOAb and TRAb were 1300 IU/mL and 12.72 IU/mL. The median thyroid iodine uptake rates at 2, 6 and 24 h were 37.25, 66.24 and 72.52%, respectively. The 6 h/24 h thyroid iodine uptake rates was 92.07%. The average dose of RAI therapy was 8.81 mCi. Overall, as shown in Fig. 1, the total cure rate of RAI therapy of GD (including euthyroid patients and early hypothyroid patients) was 72.76%. Nine patients (2.88%) achieved euthyroidism, and early hypothyroidism occurred in 218 (69.87%) patients. The 131 I therapy was ineffective in 85 patients (27.24%). In 85 patients who failed the first time RAI, 84 underwent a second RAI treatment and all of them became permanently hypothyroid. Only 1 patient underwent a thyroidectomy and was euthyroid after 6 months’ follow-up.

**Table 1:** The basic clinical characteristics of patients in this study

Variables	Range
Male: Female	54 (17.3%): 258 (82.7%)
Age (years)	45.07 ± 12.79
Duration of Graves’ hyperthyroidism (months)	6 (1, 376)
Thyroid weight (g)	52.21 (19.12, 239.00)
ATD therapy history (Male: Female)	42 (77.8%): 205 (80.7%)
Propyltiouracil (Male: Female)	5 (11.9%): 26 (12.7%)
Methimazole (Male: Female)	37 (88.1%): 179 (87.3%)
FT3 (pmol/L)	22.08 (4.35, 36.70)
FT4 (pmol/L)	51.68 (3.51, 154.80)
TSH (uIU/mL)	0.005 (0.001, 0.250)
TPOAb (IU/mL)	1300 (23.30, 1300.00)
TRAb (IU/mL)	12.72 (0.30, 40.00)
2-h RAIU	37.25 (7.80, 83.69)
6-h RAIU	66.24 (14.40, 95.62)
24-h RAIU	72.52 (27.29, 99.90)
6/24-h uptake ratio	92.07 (49.48, 126.70)
Dose of RAI (mCi)	8.81 ± 2.90

*FT3* Free triiodothyronine; *FT4* Free thyroxine; *TSH* Thyrotropin; *TPOAb* Thyroperoxidase antibody; *TRAb* Thyrotropin receptor antibody; *RAIU* Radioactive iodine uptake

### Explore the risk factors affecting the occurrence of early hypothyroidism

**Fig. 2:**
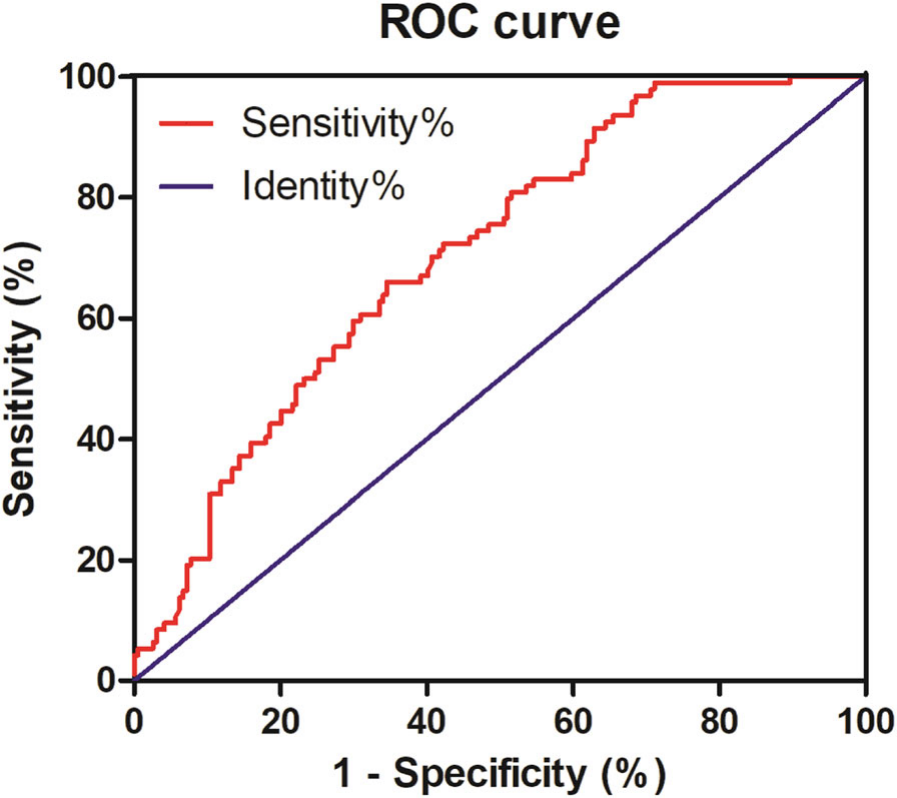
The male gender, smaller thyroid weight and lower 6-h RAIU three-factors combined ROC curve analysis for early hypothyroidism

As shown in Table 2, there were 94 patients with non-hypothyroidism, including 4 males and 90 females; 218 patients with early hypothyroidism, including 50 males and 168 females. Gender, duration of disease, thyroid weight, 2-h RAIU, 6-h RAIU, 24-h RAIU, 6/24-h uptake ratio and dose of RAI were significantly different between non-hypothyroid group and hypothyroid group. As shown in Table 3, taking the incidence of hypothyroidism as the dependent variable, a binary logistic regression was performed, and the results showed that male gender, smaller thyroid weight and lower 6-h RAIU were associated with early hypothyroidism. At last, multi-factors combined ROC curve analysis suggested that the predictive power of male gender, smaller thyroid weight and lower 6-h RAIU for early hypothyroidism was 0.711 (Fig. 2).

**Table 2:** The comparation of early hypothyroid risk factors between non-hypothyroid group and hypothyroid group

Variables	Non-hypothyroid	Hypothyroid	***P*** value
Male: Female	4: 90	50: 168	0.001
Age (years)	43.82 ± 13.17	45.60 ± 12.63	0.261
Duration of Graves’ hyperthyroidism (months)	12 (1, 240)	6 (1, 376)	0.019
ATD therapy history: Non-ATD therapy history	63: 20	184: 45	0.393
Thyroid weight (g)	60.83 (23.02, 239.00)	49.82 (19.12, 163.3)	0.001
FT3 (pmol/L)	22.48 (4.68, 36.70)	21.33 (4.35, 36.69)	0.341
FT4 (pmol/L)	57.75 (3.51, 154.80)	49.55 (13.04, 154.80)	0.091
TSH (uIU/mL)	0.005 (0.001, 0.100)	0.005 (0.001, 0.250)	0.304
TPOAb (IU/mL)	1300 (23.30, 1300.00)	1300 (27.00, 1300.00)	0.514
TRAb (IU/mL)	13.56 (0.30, 40.00)	12.72 (0.30, 40.00)	0.601
2-h RAIU	39.86 (7.80, 83.69)	35.49 (9.01, 81.98)	0.009
6-h RAIU	70.38 (14.40, 95.62)	65.41 (16.53, 95.51)	0.001
24-h RAIU	74.20 (29.10, 99.90)	71.37 (27.29, 97.11)	0.008
6/24-h uptake ratio	94.59 (49.48, 115.87)	90.94 (51.77, 126.70)	0.015
Dose of RAI (mCi)	9.48 ± 3.08	8.52 ± 2.78	0.007

*FT3* Free triiodothyronine; *FT4* Free thyroxine; *TSH* Thyrotropin; *TPOAb* Thyroperoxidase antibody; *TRAb* Thyrotropin receptor antibody; *RAIU* Radioactive iodine uptake

**Table 3:** Logistic analysis of the risk factors of early hypothyroid

						95% CI		
Variables	B	S.E.	Wald	***P***	OR	Lower	Upper	
Gender	2.087	0.569	13.457	0.001	8.061	2.643	24.582	
6 h thyroid iodine uptake rate (%)	0.019	0.009	4.590	0.032	1.019	1.002	1.037	
Thyroid weight (g)	0.020	0.006	12.931	0.001	1.020	1.009	1.031	
Constant	−7.236	1.329	29.653	0.001	0.001	–	–	

*OR* Odds ratio; *CI* Confidence interval

## Discussion

RAI therapy for the treatment of GD has been used since the 1940s [16] and now it is still one of the most important treatments for GD [17, 18]. In most countries around the world, a fixed dose of iodine 131 is generally used for treatment. Both euthyroidism and hypothyroidism are considered a successful therapy. However, the RAI use is decreasing even in the United States in the past decade, partly because a preference to avoid hypothyroidism and lifelong hormone replacement [19]. In this study, we use the calculated dose method to determine the dose of iodine 131, and try to find the influence factors of hypothyroidism during RAI treatment.

It has been reported that the majority of GD occurs between 30 and 60 years old, most of them were females, with a male to female ratio of about 1:3–1:10 [1, 2, 9]. Our study showed that the average age of the patients was 45.07 ± 12.59 years and the ratio of male to female was 1:4.78, which was similar to the previous reports. In the literatures, the incidence of hypothyroidism after RAI therapy ranged from 8.5 to 90% [20–24]. Our results indicated that the early hypothyroidism occurred in 218 (69.87%) patients, and only nine patients (2.88%) achieved euthyroidism, which was consistent with a recent study conducted in Singapore [25].

**Fig. 3:**
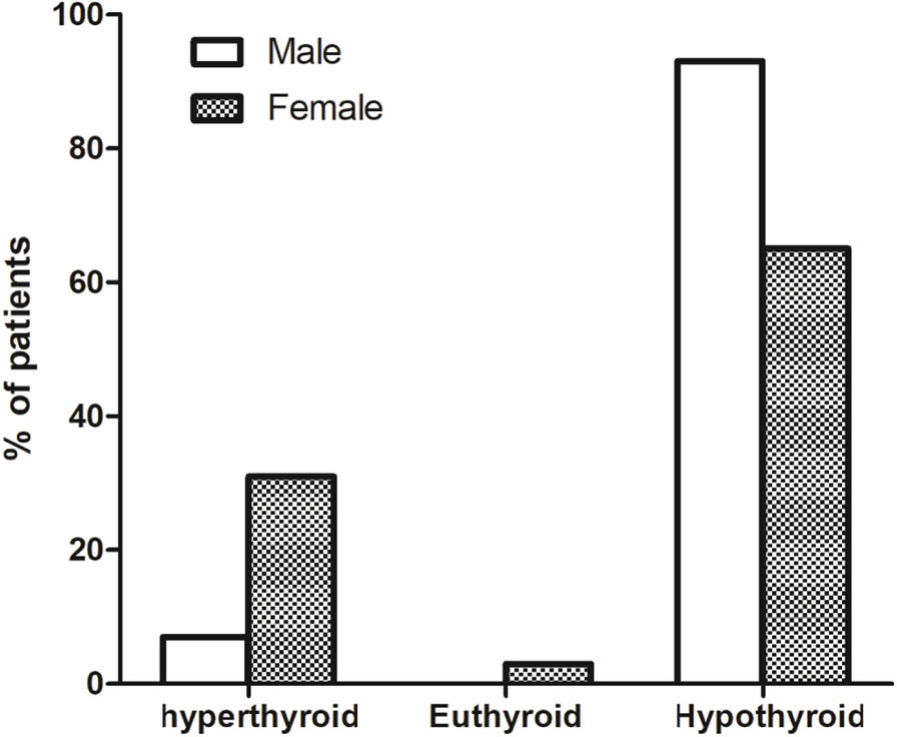
The thyroid function of the patients six months after RAI treatment

**Fig. 4:**
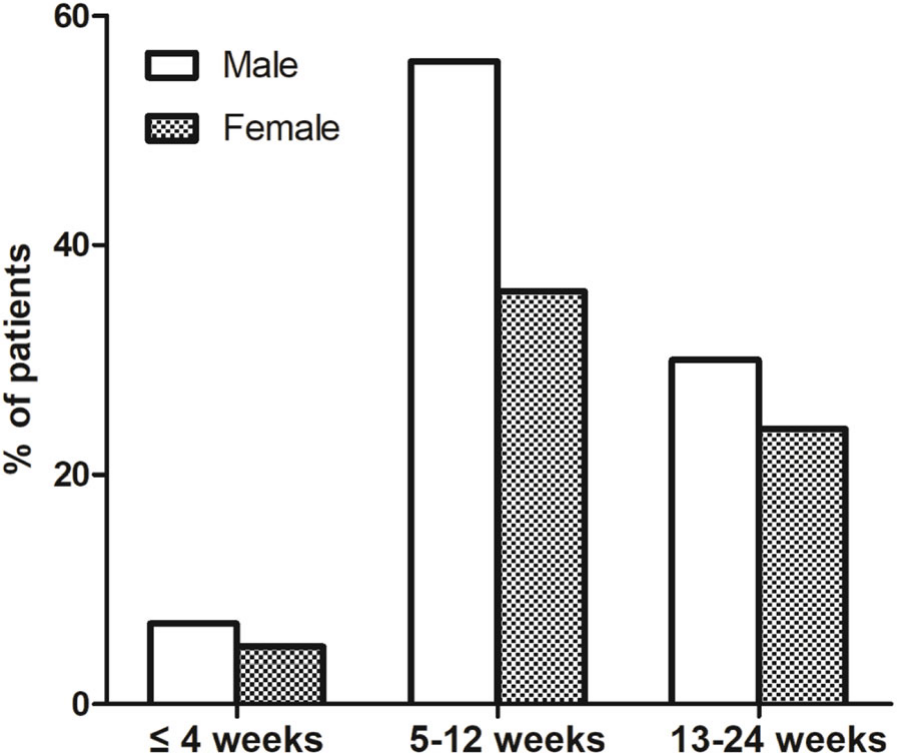
The occurrence of hypothyroidism after RAI treatment within six months

Most of the previous studies suggested that there were no differences in the incidence of hypothyroidism between men and women after RAI treatment. However, some studies have suggested that female gender to be an independent predictor of hypothyroidism [26, 27]. In our study, as shown in Fig. 3 and Figs. 4, 50 of 54 male patients occured early hypothyroidism, but only 168 of the 258 female patients had early hypothyroidism. More than 50% male patients became hypothyroid in 4–12 weeks after RAI therapy, but only 36% female patients became hypothyroid at that time. There was a significant statistical difference between men and women. The subsequent logistic regression analysis also suggested that male gender was a risk factor of early hypothyroidism even accounting for other clinical factors. This is quite different with the previous studies, but we found recently a research of Malaysia also prompt that men became to hypothyroid easier than women [28]. We hypothesized that the difference may be because the genetic susceptibility among different races.

De Jong et al. believed that larger thyroid volumes (> 50 mL) and increasing 5/24-h 131I turnover (≥0.8) are independent risk factors of hypothyroidism [13]. Yang et al. showed that RAI treatment was more successful in patients with lower weight of the thyroid [29]. We found that the non-hypothyroid patients had a median thyroid weight of 60.83 g, but the hypothyroid patients only 49.82 g. We also found the non-hypothyroid patients had higher 2 h, 6 h, 24 h thyroid iodine uptake rate and 6 h/24 h thyroid iodine uptake rate than hypothyroid patients (Table. 2), this is consistent with previous researches.

Some studies demonstrated that FT3 level, FT4 level, TSH level and TRAb titre before RAI treatment were significantly associated with treatment failure of RAI [30, 31], but we didn’t find the associations in our study. Perhaps because these factors are too weak to affect the occurrence of hypothyroid.

## Conclusions

In short, our study demonstrated that male gender, 6 h thyroid iodine uptake rate and thyroid weight are associated with early hypothyroid after RAI therapy. Adjust the treatment dose in these patients may decrease the occurrence of early hypothyroidism and improve the outcome of RAI therapy.

## Data Availability

The data used to support the findings of this study are available from the corresponding author upon request.

## Abbreviations

ATD: Anti-thyroid drugs
FT3: Free triiodothyronine
FT4: Free thyroxine
GD: Graves’ disease
RAI: Radioactive iodine
RAIU: Radioactive iodine uptake
TPOAb: Thyroperoxidase antibody
TRAb: Thyrotropin receptor antibody
TSH: Thyrotropin
